# Species limits in polymorphic mimetic *Eniclases* net-winged beetles from New Guinean mountains (Coleoptera, Lycidae)

**DOI:** 10.3897/zookeys.593.7728

**Published:** 2016-05-26

**Authors:** Matej Bocek, Ladislav Bocak

**Affiliations:** 1 Laboratory of Molecular Systematics, Department of Zoology, Faculty of Science, Palacky University, 17. Listopadu 50, 771 46 Olomouc, Czech Republic

**Keywords:** Aposematism, bPTP model, Coleoptera, *cox1* mtDNA, genetic distance, morphology, new species, species delimitation

## Abstract

Species delimitation was compared in a group of closely related lineages of aposematically colored *Eniclases* (Coleoptera, Lycidae) using morphology, genetic distances, and Bayesian implementation of the Poisson Tree Processes model. A high diversity of net-winged beetles was found in previously unsampled regions of New Guinea and ten new species are described: *Eniclases bicolor* **sp. n.**, *Eniclases bokondinensis* **sp. n.**, *Eniclases brancuccii* **sp. n.**, *Eniclases elelimensis* **sp. n.**, *Eniclases infuscatus* **sp. n.**, *Eniclases niger* **sp. n.**, *Eniclases pseudoapertus* **sp. n.**, *Eniclases pseudoluteolus* **sp. n.**, *Eniclases tikapurensis* **sp. n.**, and *Eniclases variabilis* **sp. n.** Different levels of genetic and morphological diversification were identified in various sister-species pairs. As a result, both morphological and molecular analyses are used to delimit species. Sister-species with uncorrected pairwise genetic divergence as low as 0.45% were morphologically distinct not only in color pattern, but also in the relative size of eyes. Conversely, differences in color pattern regardless of their magnitude did not necessarily indicate genetic distance and intraspecific mimicry polymorphism was common. Additionally, genetic divergence without morphological differentiation was detected in one sister-species pair. Low dispersal propensity, diverse mimicry patterns, and mimetic polymorphism resulted in complex diversification of *Eniclases* and uncertain species delimitation in recently diversified lineages.

## Introduction

The Papuan beetle fauna remains one of the most under-investigated despite high species richness in the Oceanian region and a long history of taxonomic research in Papua (e.g. Kleine 1926). Recent studies show exceptional diversity (Riedel et al. 2013, Toussaint et al. 2014). Previous reports on Papuan net-winged beetles included chaotic classification and poor species delimitation resulting from formal morphological descriptions using a semaphoront without diagnostic characters. DNA data provide a tool to accelerate biodiversity research, but these data must be viewed as part of a process of cross-validation of hypotheses on species limits based on both morphology and genetic information (e.g. Meyer and Paulay 2005, Meier et al. 2006, Baselga et al. 2013, Jorger and Schrodl 2013). Here, we present a taxonomic study dealing with *Eniclases* Waterhouse, 1879 (Metriorrhynchini), a genus in which most species are aposematically colored. They belong to numerous mimetic complexes, mostly formed by net-winged beetles, which are unpalatable (Bocak and Yagi 2010). Additionally, the net-winged beetles have weak dispersal ability and consequently prone to rapid differentiation of local populations (Li et al. 2015).

Most Papuan net-winged beetles belong to genera known from Australia (Calder 1998, Sklenarova et al. 2014), but some including *Eniclases*, are endemic. These lineages diversified probably in New Guinea and adjacent islands (Sklenarova et al. 2013) and are diverse despite the supposedly short existence of New Guinea in its present form. New Guinea is a young landmass resulting from uplift of the northern margin of the Australian tectonic plate and accretion of oceanic islands about 5 million years ago (Hill and Hall 2002). *Eniclases* was revised by Bocak and Bocakova (1991), including valid 27 species. The morphology-based revision of Bocak and Bocakova (1991) primarily investigated material from the eastern part of New Guinea. The fauna of Western New Guinea remained poorly studied with few species reported from the lowlands at the northern coast (4 spp.), the Panai Lake region (3 spp.), the Fak Fak Peninsula (2 spp.), and the Star Mts. (Oksibil area, 2 spp.). The faunas of the Central Mts. region and the Bird’s Head Peninsula were unknown.

A taxonomic study based on material representing *Eniclases* from the western part of the island, mainly from the Central Mts, is presented. The aim was to compare species delimitations inferred from genetic distances (barcoding approach; Hebert et al. 2003, Meier et al. 2006) and the tree shape (the Bayesian Poisson Tree Processes method, bPTP; Zhang et al. 2013). The status of these putative species inferred from mtDNA data was tested by the presence of morphological characters. Specifically, we investigate intraspecific color polymorphism and morphological diagnostic characters. Descriptions and diagnoses of new species are presented using available evidence and possible scenarios for diversification of *Eniclases* are discussed.

## Methods

### Material and laboratory procedures

**Figures 1–2.**
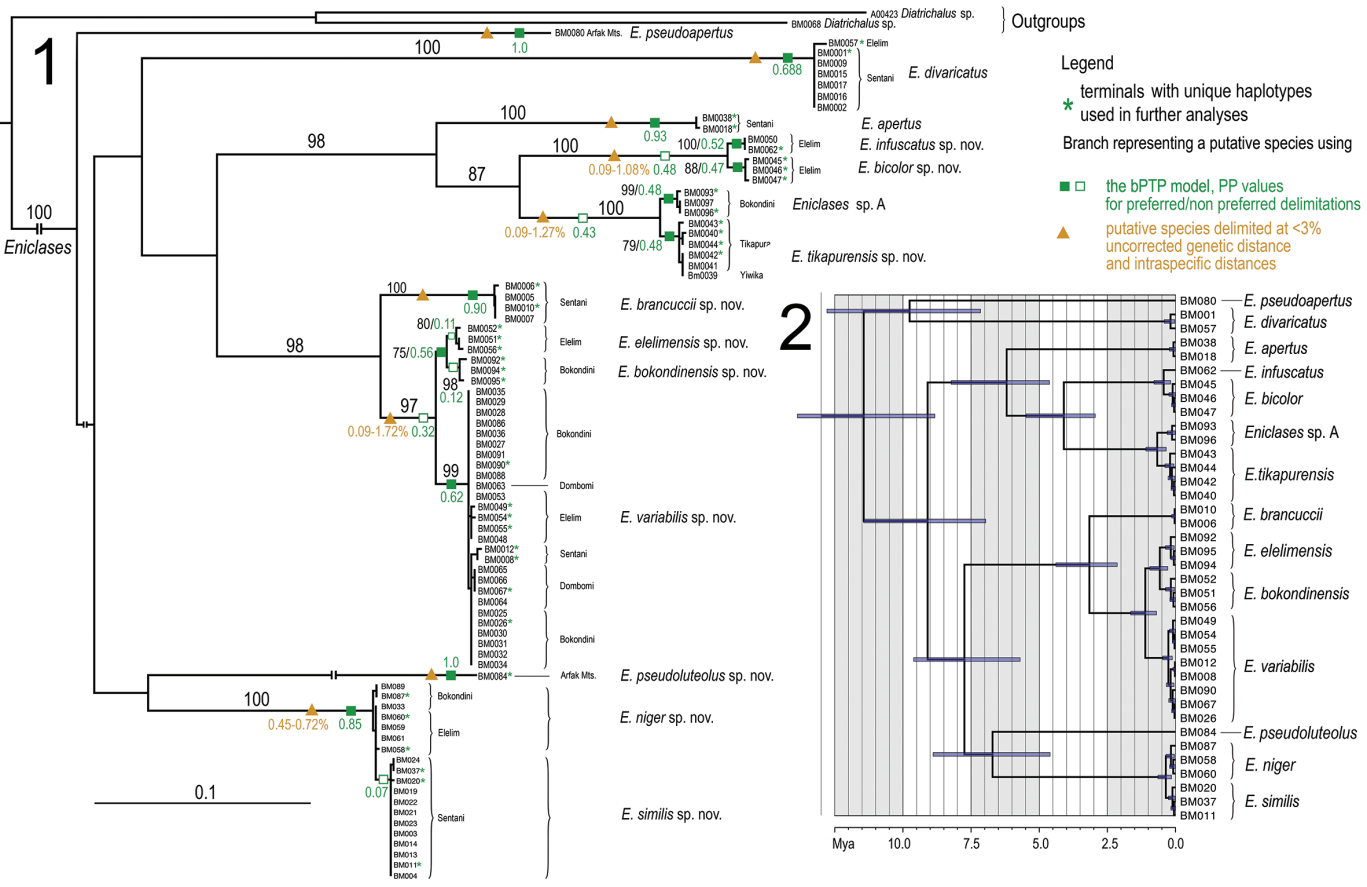
**1** Phylogenetic tree of *Eniclases* inferred from the maximum likelihood optimality criterion; a basal part of outgroups omitted. The numbers at branches show bootstrap support greater than 50%, genetic divergence within respective putative species and posterior probabilities inferred from the bPTP model **2** Dated tree produced using Bayesian inference.

In total, 81 specimens of *Eniclases* from the western part of New Guinea: the Central, Cyclops and Arfak Mts. were kept in -20 °C until DNA extraction. Each specimen was given a voucher number (Figs 1–2 and Taxonomy section, where exact locality data are given), voucher specimens are deposited in the collection of the Laboratory of Molecular Systematics, Olomouc and all sequences were submitted to GeneBank (Accession Numbers KT256092–172). The *cox1* + tRNA-Leu + *cox2* mtDNA fragment (hereafter referred as *cox1* only) was amplified using primers JerryM (CAACAYYTATTTTGRTTYTTTGG) and Marcy (TARTTCRTATGWRCAATAYCAYTGRTG) or JerryN (CAACAYYTATTYTGATTYTTYGG) and MarcyN (TTCRTAWGTTCARTATCATTGRTG). DNA extraction, PCR settings and cycle sequencing conditions follow Bocak and Bocakova (2008). The PCR products were purified using PCRμ96™ Plates (Millipore Inc.) and sequenced by an ABI 3130 automated sequencer using the BigDye® Terminator Cycle Sequencing Kit 1.1.

### Phylogenetic analyses and species delimitation

Sequences were edited using the Sequencher 4.8 software package (Gene Codes Corp.) and combined with homologous sequences of 25 outgroup taxa representing Cautirina and Metriorrhynchina (all sequences taken from GenBank). The length invariable *cox1* mtDNA fragment was aligned using ClustalX 2.1 (Thompson et al. 1997) under default parameters and the phylogenetic analysis was carried out under the maximum likelihood criterion using RAxML 7.3.1 (Stamatakis 2006) and the GTR + I + G model for all partitions identified by jmodeltest 2.1.7 (Darriba et al. 2012). All genes and codon positions in the protein coding fragments were partitioned and parameters unlinked for each partition. Bootstrap values (BS) were assessed by analyzing 1000 pseudoreplicates using the rapid bootstrap algorithm under the GTRCAT model (Stamatakis et al. 2008). Trees were edited and visualized in Dendroscope 3.1 (Huson et al. 2007). In order to estimate the origin of closely related lineages with different mimetic patterns, the tree was dated using Beast 1.8.1 (Drummond et al. 2012) with the same model as in the maximum likelihood analysis. As there is no fossil record of metriorrhynchine Lycidae, we relied on the molecular evolution rates used earlier and tested two rates, 0.0115 substitutions per lineage per million years (s/l/my, Brower 1994), which gave results in agreement with tectonics in earlier study on *Metriorrhynchus* (Bocak and Yagi 2010) and the rate 0.0177 s/l/my calculated by Papadopoulou et al. (2010) for Tenebrionidae. We used the uncorrected lognormal clock model, Speciation: Birth Death, and sampled every 1,000 generations from a 10 million generation MCMC chain. The first 4 million generations were discarded as burn-in after evaluation of likelihood values and the effective sample size >1000 in Tracer 1.6 (Rambaut et al. 2013).

The genetic intra- and interspecific differentiation among whole-fragment sequences was estimated using Kimura 2-parameter genetic distances in MEGA6 (Tamura et al. 2013) and clusters of similar sequences were identified in Species Identifier 1.7.7 using a 3% barcoding threshold (Meier et al. 2006). The densities for intra- and interspecific differences were visualized in R (http://r-project.org). Further, species level entities were defined using the maximum likelihood and Bayesian implementation of the Poisson Tree Processes (bPTP) model for species delimitation (the bPTP server at species.h-its.org; Zhang et al. 2013).

Morphological characters were observed for all sequenced specimens: measurements of the body size, maximum diameter of eyes in the lateral view, the minimum interocular distance in the frontal part of cranium, color patterns of the pronotum and elytra, shape of pronotum and antennae, and structure of the elytral costae. Photographs were taken using a binocular microscope Olympus SZX-16 and were assembled in Helicon Focus 6 (www.heliconsoft.com). Due to previously reported uniformity of the genitalia of both sexes (Bocak and Bocakova 1991), those of only a few species were illustrated.

### Abbreviations

DEIM
 Deutsches Entomologisches Institute, Müncheberg, Germany 


MHNP
 Muséum national d’Histoire naturelle, Paris, France 


LMBC
 Voucher collection, Department of Zoology, UP Olomouc, Czech Republic 


## Results

### Molecular phylogeny

The 1101 bp fragment of mtDNA was sequenced for 81 individuals of *Eniclases* from western New Guinea. The DNA fragment consisted of 782 base pairs (bp) *cox1* mtDNA, 59 bp tRNA-Leu, and 260 bp *cox2* mtDNA. We identified 38 unique haplotypes and the Kimura-2-parameter genetic distances among *Eniclases* sequences spanned 0.09–14.31%. The maximum likelihood analysis produced the tree in Fig. 1. *Eniclases* formed a monophylum with 100% bootstrap support and the fauna of the Central Mts. was represented by four deeply rooted clades of closely related species (Fig. 1). The dated tree inferred with 0.0115 s/l/my rate is shown in Fig. 2 and suggested that closely related species pairs originated within in the last million years. The higher rate of 1.77% produced even shallower splits for the terminal lineages (results not shown).

The genetic divergence between all haplotypes was evaluated and the highest densities were between 0.0–2.0% and 9.0–14% (Fig. 4). Further, morphological divergence was considered within genetically close terminals and three morphologically distinguishable species pairs were identified: *Eniclases elelimensis* and *Eniclases bokondinensis* (mean interspecific K-2-P distance 0.79%, maximum intraspecific distance 0.17%); *Eniclases infuscatus* and *Eniclases bicolor* (1.07% and 0.12%); *Eniclases niger* and *Eniclases similis* (0.59% and 0.16%). Relatively low divergence was found between *Eniclases variabilis* and *Eniclases elelimensis* (<1.59%), *Eniclases variabilis* and *Eniclases bokondinensis* (<1.72%) and between *Eniclases brancuccii* and three species in its sister clade (3.90–4.59%). The clade of *Eniclases tikapurensis* and *Eniclases* sp. A consisted of two sister-subclades with inter-clade genetic difference 1.12–1.28% (Fig. 1) and these candidate species were morphologically indistinguishable.

**Figures 3–5.**
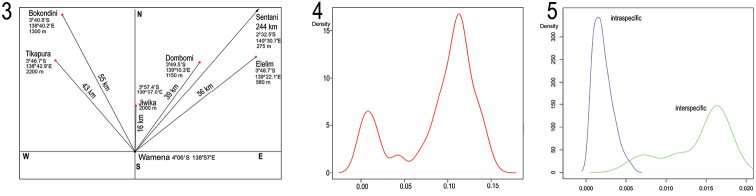
**3** The positions, coordinates and elevations of sampled localities in the Central Mountains of New Guinea **4** Density plots of genetic distances of all *Eniclases* samples **5** Density plots of intra- and interspecific genetic distances between pairs of closely related species of *Eniclases* (*Eniclases infuscatus* and *Eniclases bicolor*; *Eniclases tikapurensis* and *Eniclases* sp. A; *Eniclases variabilis*, *Eniclases elelimensis* and *Eniclases bokondinensis*; *Eniclases niger* and *Eniclases similis*).

Furthermore, putative species were identified using genetic distance and the phylogenetic tree. The pairwise differences among *cox1* mtDNA sequences merged haplotypes in 9 clusters when the threshold 3% was applied. The inferred clusters merged *Eniclases infuscatus* and *Eniclases bicolor*, *Eniclases tikapurensis* + *Eniclases* sp. A, the clade *Eniclases elelimensis* + *Eniclases bokondinensis* + *Eniclases variabilis* and the pair *Eniclases niger* + *Eniclases similis* (Fig. 1, the clades labeled with triangles and intra-clade genetic distances). Most species differed in coloration, often additionally in eye size and geographic origin (see taxonomy section for detailed morphological descriptions). The maximum likelihood PTP method proposed identical species limits as the distance approach (results not shown). Unlike these, the Bayesian implementation of the PTP model suggested 9–19 putative species, mean 11.97 species. The most supported partitions, considered further as putative species, are displayed in Figure 1 (12 putative species designated by squares and labels designating posterior probabilities, filled green squares designate partitions with the highest PPs, empty squares suboptimal, non-preferred partitions). The posterior delimitation probabilities (PP) were high only for *Eniclases apertus* and *Eniclases brancuccii* partitions (≥90%); other clades obtained moderate to very low PPs. On the other hand, the alternative species delimitations corresponding to those from distance analyses and delimitations based on morphological traits had even lower PPs (Fig. 1).

Fourteen species were identified in the sequenced material, including ten new to science and described in the Taxonomy section. The delimitation of species was based on morphological characters (the size of eyes, shape of male antennae and coloration), genetic distance, and delimitations of putative species inferred from the bPTP model.

### Taxonomy

#### Eniclases

Taxon classificationAnimaliaColeopteraLycidae

Waterhouse, 1879

Eniclases Waterhouse, 1879: 66.

##### Type species.

*Lycus luteolus* Waterhouse, 1878 by monotypy.

##### Diagnosis.

*Eniclases* is similar to *Trichalus* Waterhouse, 1877 and they share a small to medium sized, dorso-ventrally flattened body; a characteristic shape of a pronotum with acutely projecting posterior angles and a hump in a posterior third of a pronotal margin; nine elytral costae in the humeral part of elytra (four robust primary costae and five weak secondary costae) and a shortened primary costa 1 (Figs 6–50). Unlike *Trichalus*, the median pronotal areola is absent in *Eniclases* and only two divergent longitudinal keels are present in the pronotum (Figs 6–29). Additionally, all *Eniclases* have a characteristic cap-shaped apex of the phallus (Figs 43–50) similar to those of *Schizotrichalus* Kleine, 1926 (Bocak 2002). The detailed redescription of *Eniclases* was published by Bocak and Bocakova (1991).

**Figures 6–17.**
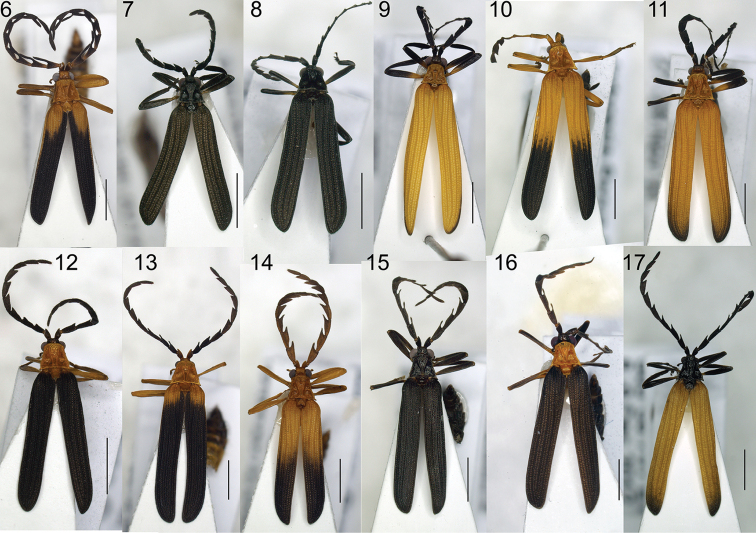
Habitus of *Eniclases*: **6** *Eniclases divaricatus*, male **7** *Eniclases pseudoapertus* sp. n., male **8** *Eniclases apertus*, male **9** *Eniclases tikapurensis* sp. n., male **10** *Eniclases bicolor* sp. n., female **11** *Eniclases infuscatus* sp. n., female **12** *Eniclases brancuccii* sp. n., female **13, 14** *Eniclases similis*, male **15, 16** *Eniclases niger* sp. n., male **17** *Eniclases bokondinensis* sp. n., female. Scale bars: 2 mm.

**Figures 18–29.**
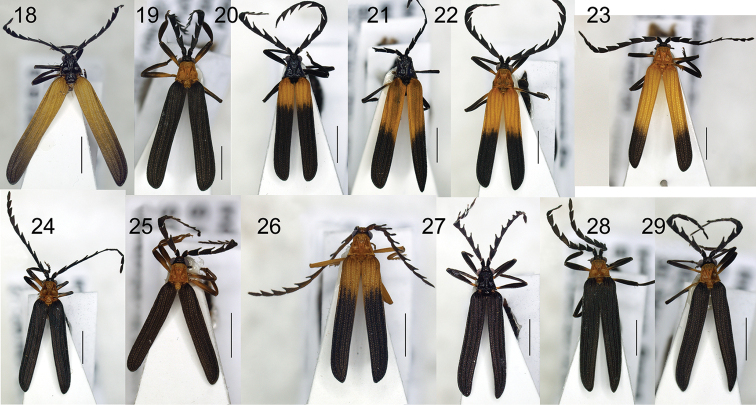
Habitus of *Eniclases*: **18** *Eniclases bokondinensis* sp. n., female **19** *Eniclases elelimensis*, female **20–29** *Eniclases variabilis* sp. n. Scale bars: 2 mm.

##### Phylogenetic relationships.

The shape of the pronotum, shortened elytral costa one, and shape of male genitalia (Figs 6–50) support close relationships of *Eniclases* and other trichaline genera. At present, the trichaline lineages form a subordinate clade within Metriorrhynchina and consist of *Eniclases*, *Flabellotrichalus* Pic, 1921, *Microtrichalus* Pic, 1921, *Schizotrichalus* Kleine, 1926, and *Trichalus* Waterhouse, 1877 (Bocak 2002, Sklenarova et al. 2014).

**Figures 30–50.**
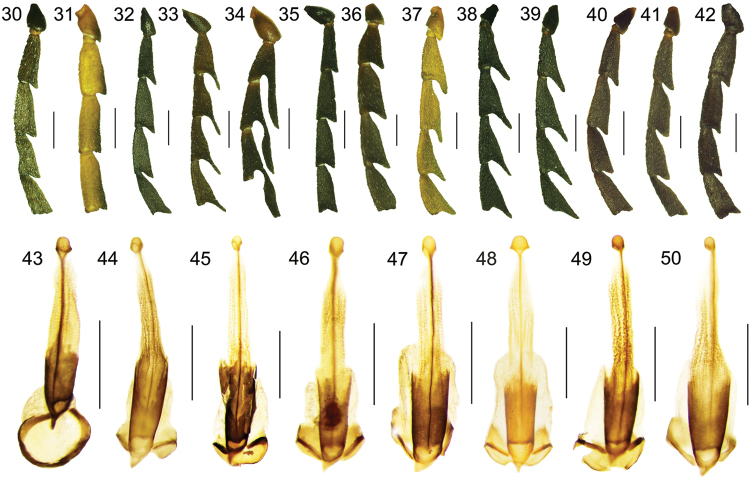
Basal antennomeres of (Figs **30–42**): **30** *Eniclases apertus*, male **31** *Eniclases bicolor* sp. n., female **32** *Eniclases bokondinensis* sp. n., female **33** *Eniclases brancuccii* sp. n., male **34** *Eniclases divaricatus*, male **35** *Eniclases infuscatus* sp. n., female **36** *Eniclases pseudoluteolus*, male **37** *Eniclases similis*, male **38** *Eniclases variabilis* sp. n., male **39** *Eniclases elelimensis*, male **40** *Eniclases niger* sp. n., female **41** *Eniclases tikapurensis*, male **42** *Eniclases pseudoapertus* sp. n., male. Male genitalia, ventral view (Figs **43–50**): **43** *Eniclases pseudoapertus* sp. n. **44** *Eniclases divaricatus* **45** *Eniclases tikapurensis* sp. n. **46** *Eniclases brancuccii* sp. n. **47** *Eniclases variabilis* sp. n. **48** *Eniclases pseudoluteolus* sp. n. **49** *Eniclases niger* sp. n. **50** *Eniclases similis*. Scale bars: 0.5 mm.

#### Eniclases pseudoapertus sp. n.

Taxon classificationAnimaliaColeopteraLycidae

http://zoobank.org/14959462-5DD6-4728-AB10-A2056B6CEB1E

Figs 7 , 42

##### Material examined.

Holotype. Male (Voucher number BM0080), Indonesia, Irian Jaya, Arfak Mts., Maibri village, 1570 m, Nov.–Dec. 1991 (LMBC).

##### Diagnosis.

*Eniclases pseudoapertus* resembles *Eniclases apertus* Pic, 1923 in the small body and black coloration (Fig. 7); *Eniclases apertus* has smaller eyes than *Eniclases pseudoapertus* (Tab. 1).

**Table 1.** Measurements of *Eniclases* spp. (all measurements in mm, n.a. – not available).

	Body length	Width humeri	Pronotum		Eye diameter/distance	
			length	width	male	female
*Eniclases pseudoapertus*	6.3	1.6	0.75	1.2	1.4	n.a.
*Eniclases divaricatus*	8.8–9.7	2.1–2.3	1.2–1.3	1.7–1.7	0.92–0.96	0.80–0.87
*Eniclases pseudoluteolus*	9.3	2.3	1.15	1.6	0.9	n.a.
*Eniclases apertus*		5.8–6.9	1.4–1.7	0.9	1.25	1.15–1.17
*Eniclases tikapurensis*	9.5–11.1	2.0–2.5	1.1–1.3	1.4–1.7	1.11–1.40	0.92–1.06
*Eniclases bicolor*	10.3	2.4	1.4	1.7	n.a.	0.71–0.74
*Eniclases infuscatus*	12.1	2.5	1.25	1.6	n.a.	0.79–0.84
*Eniclases brancuccii*	7.6–8.0	1.8–1.9	1.0–1.1	7.5–8.0	1.00	0.84–0.91
*Eniclases bokondinensis*	9.2	2.05	1.0	1.35	n.a.	0.72–0.82
*Eniclases elelimensis*	6.9–8.1	1.5–1.9	0.9–1.1	1.3–1.4	n.a.	0.78–0.89
*Eniclases variabilis*	6.6–8.2	1.6–2.0	0.1–1.1	1.1–1.35	0.83–0.95	0.70–0.85
*Eniclases niger*	9.2–11.6	2.2–2.8	1.3–1.6	9.0–11.5	1.17–1.28	0.89
*Eniclases similis*	7.5–9.7	1.9–2.3	1.1–1.4	1.8–1.8	1.02–1.15	0.89

##### Description.

Male. Body length 6.3 mm, uniformly dark colored, only trochanters and bases of femora light brown (Fig. 7). Head with large hemispherically prominent eyes, their maximum diameter 1.40 times minimal interocular distance. Antennae slender, serrate, almost parallel-sided, with short apical process of antennomere 3 (Fig. 42). Pronotum transverse, with almost straight sides, elytra with weak secondary costae and irregular cells.

##### Distribution.

Indonesia, Arfak Mts.

##### Etymology.

The specific name refers to similarity with *Eniclases apertus*.

#### Eniclases divaricatus

Taxon classificationAnimaliaColeopteraLycidae

(Pic, 1921)

Figs 6 , 34

Trichalus (Trichalolus) divaricatus Pic, 1921: 10.

##### Material examined.

Lectotype. Female, New Guinea, Humboldt Bay, Doherty lgt., coll. Pic (MHNP). Other material examined. 4 males, 2 females (BM0001–2, 9, 15–17), Indonesia, Irian Jaya, Sentani, Cyclops Mts., 300 m, Nov.–Dec. 1991; female (BM0057), Indonesia, Irian Jaya, Elelim, path to Apalapsili, 600 m, Nov.–Dec. 1991 (LMBC).

##### Diagnosis.

*Eniclases divaricatus* is the only Papuan species with the bicolored elytra and flabellate male antennae (Figs 6, 34). Additionally, this species has the characteristic pattern of bright humeri and dark colored suture, which is shared only with some specimens of *Eniclases similis* from the same locality (Figs 6, 13). The similarly colored females cannot be distinguished as their relative size of eyes is similar (Table 1).

##### Redescription.

Male. Body length 8.8–9.7 mm. Head, thorax, legs, and humeri yellow to orange, antennae except basal part, abdomen, and most of elytra dark colored. Head with moderately large, hemispherically prominent eyes, their diameter 0.92–0.96 times minimum interocular distance Antennae flabellate, lamella of antennomere 3 slightly longer than the body of antennomere, other lamellae similar (Fig. 34). Pronotum transverse, with almost straight, slightly elevated lateral margins, elytra with quite strong straight secondary costae and regular dense cells (Fig. 6).

##### Distribution.

Central North New Guinea.

#### Eniclases infuscatus sp. n.

Taxon classificationAnimaliaColeopteraLycidae

http://zoobank.org/AF02A670-1C21-49A3-A7F3-5B7C35F494C8

Figs 11 , 35

##### Material examined.

Holotype. Female (BM0050), Indonesia, Irian Jaya, Bokondini, 1300 m, Nov.–Dec. 1991 (LMBC). Paratype. Female (BM0062), Indonesia, Irian Jaya, Elelim, path to Apalapsili, 600 m, Nov.–Dec. 1991 (LMBC).

##### Diagnosis.

*Eniclases infuscatus* has a unique color pattern among western Papuan *Eniclases*. The upper part of the body is yellow to orange and only tips of elytra and the posterior part of the lateral margins are infuscate. This species partly resembles in the coloration *Eniclases tikapurensis*, which is slender and pale colored (Figs 9, 11).

##### Description.

Female. Body length 12.1 mm, robust. Head brown, abdomen, meso- and metathorax dark colored, most of legs similarly colored, only trochanters and basal half of femora light brown; pronotum and elytra yellow to orange, only tips and posterior margins of elytra infuscate, transition between dark and bright parts of elytra gradual (Fig. 11). Head with small hemispherically prominent eyes, their diameter 0.79–0.84 times minimum interocular distance. Antennae serrate, antennomeres 3–4 triangular, antennomeres 5–10 almost parallel-sided (Fig. 35). Pronotum transverse, with almost straight lateral margins, elytra with weak, regular secondary costae and regular, often transverse, small cells.

##### Distribution.

New Guinea, Bokondini region.

##### Etymology.

The specific name refers to blackened edge of the apical part of elytra.

#### Eniclases bicolor sp. n.

Taxon classificationAnimaliaColeopteraLycidae

http://zoobank.org/5F220BB6-75C7-4C6B-8996-822824762A3A

Figs 10 , 31

##### Material examined.

Holotype. Female (BM0046), Indonesia, Irian Jaya, Elelim, path to Apalapsili, 600 m, Nov.–Dec. 1991 (LMBC). Paratypes. 2 females (BM0045, 47), same locality data as the holotype (LMBC).

##### Diagnosis.

*Eniclases bicolor* resembles some forms of *Eniclases similis* from the Cyclops Mts. and differs in a higher contrast between light colored costae and dark cells in a transitional area between the dark and light parts of their elytra (Fig. 10). Additionally, the females of *Eniclases bicolor* have small eyes (Table 1).

##### Description.

Female. Body length 10.3 mm, robust. Head, basal part of antennae, pro- and mesothorax, basal half of elytra and legs yellow to light orange, apical half of antennae, metathorax, half of elytra and abdomen dark, transition between dark and bright parts abrupt(Fig. 10). Head with small, hemispherically prominent eyes, their diameter 0.71–0.74 times minimum interocular distance, antennae slender, antennomeres 3–5 serrate, antennomeres 6–10 almost parallel-sided (Fig. 31). Pronotum transverse, with almost straight lateral margins and prominent posterior angles, elytra with weak, but straight secondary costae and regular, often transverse, small cells.

##### Distribution.

New Guinea, Elelim region.

##### Etymology.

The specific name refers to the coloration of elytra.

#### Eniclases tikapurensis sp. n.

Taxon classificationAnimaliaColeopteraLycidae

http://zoobank.org/CF8E0FB2-3F8F-4D6B-8AA3-3430D281B886

Figs 9 , 41 , 45

##### Material examined.

Holotype. Male (BM0039), Indonesia, Irian Jaya, Yiwika, N of Wamena, 2000 m, Nov.–Dec. 1991 (LMBC). Paratypes. 3 males, 2 females (BM0040–44), Indonesia, Irian Jaya, Tikapura village, 2200 m, Nov.–Dec. 1991 (LMBC).

##### Diagnosis.

*Eniclases tikapurensis* resembles in general appearance *Eniclases papuensis* Bocak & Bocakova, 1991 from the Panai Lake area approximately 250 km west of Yiwika. Both species are characteristic in pale hue of the yellow upper part (Fig. 9) but differ in the relative size of eyes when *Eniclases tikapurensis* has much larger eyes (Table 1).

##### Description.

Male. Body length 9.5–11.1 mm, slender (Table 1). Head, antennae, thorax, legs except trochanters and bases of femora bark brown to black, pronotum and elytra except posterior edge pale yellow (Fig. 9). Head with large, hemispherically prominent eyes, their diameter 1.11–1.40 times minimum interocular distance. Antennae serrate, antennomere 3 triangular, with pointed apical process, antennomere 4 parallel-sided in most of its length, its process shorter, shape of other antennomeres similar (Fig. 41). Pronotum transverse, lateral margins with weak bulge in basal third and prominent posterior angles, elytra with weak but well developed, straight secondary costae and regular, mostly quadrate, small elytral cells.

##### Distribution.

New Guinea, Upper Baliem Valley.

##### Etymology.

The specific name refers to the type locality, the village Tikapura, north of Tagime.

##### Remark.

The genetically distant population from Bokondini is a sister to *Eniclases tikapurensis*, but does not differ in any morphological character. As their delimitation would be based only on mtDNA sequence and further information on nuclear markers and geographical distribution is needed for these two putative cryptic species, we postpone the formal description of the population from Bokondini. The sequenced specimens representing the Bokondini population are designated as *Eniclases* sp. A. in Fig. 1.

#### Eniclases brancuccii sp. n.

Taxon classificationAnimaliaColeopteraLycidae

http://zoobank.org/42CD1738-157D-49CA-9D1D-CFC7DE13CF13

Figs 12 , 33 , 46

##### Material examined.

Holotype. Male (BM0006), Indonesia, Irian Jaya, Sentani, Cyclops Mts., 300 m, Nov.–Dec. 1991 (LMBC). Paratypes. 3 females (BM0005, 0007, 00010), the same data as the holotype (LMBC).

##### Diagnosis.

*Eniclases brancuccii* resembles in color pattern three species: *Eniclases elelimensis*, *Eniclases niger*, and *Eniclases variabilis*. The last two of them are polymorphic and only some individuals share the color pattern with *Eniclases brancuccii* (Figs 12, 16, 24–25, 28–29). *Eniclases niger* differs in the large eyes and almost parallel-sided antennomeres 3–10 (Table 1, Figs 33–40). *Eniclases elelimensis* and *Eniclases variabilis* have similar antennae (Figs 38–39) and slightly larger eyes compared to *Eniclases brancuccii*, but reliable identification of these species can only be based on the DNA sequences. To the best of our knowledge, the similarly colored forms of these species do not occur together in a single locality.

##### Description.

Male. Body length 7.6–8.0 mm, robust, head, antennae, thorax, elytra, and abdomen dark brown to black, pronotum, scutellum, and basal parts of femora yellow to orange, apical parts of femora, tibiae, and tarsi dark brown. Head with small, hemispherically prominent eyes, their diameter equals minimum interocular distance, antennae flat, slender, acutely serrate, apical process of antennomere 3 about half length of its body, further antennomeres similar in shape. Pronotum transverse, with apparent bulge in basal third, almost parallel-sided between bulge and posterior angles, elytra with weak but regular secondary costae and mostly regular, quadrate, small cells.

##### Distribution.

New Guinea, Cyclops Mountains.

##### Etymology.

The specific name ‘*brancuccii*’ is proposed in honor of the late Michel Brancucci, a specialist in Dytiscidae and Cantharidae.

#### Eniclases elelimensis sp. n.

Taxon classificationAnimaliaColeopteraLycidae

http://zoobank.org/D0E69D4B-83FF-48C8-9394-6746DE3C28D0

Figs 19 , 39

##### Material examined.

Holotype. Female (BM0056), Indonesia, Irian Jaya, Elelim, path to Apalapsili, 600 m, Nov.–Dec. 1991 (LMBC). Paratypes. 2 females (BM0051–52), the same data as the holotype (LMBC).

##### Diagnosis.

*Eniclases elelimensis* differs from *Eniclases brancuccii* in slightly smaller eyes (Table 1), but no morphological difference has been found to discriminate this species from some similarly colored individuals of *Eniclases variabilis* (Figs 24–25, 28–29) and morphology based identification can reliably assign similarly colored specimens only to the clade of *Eniclases variabilis* and related species.

##### Description.

Male. Body length 6.9–8.1 mm, robust, head, antennae, thorax, elytra, and abdomen dark brown to black, pronotum and femora yellow to light orange, apical part of femora, tibiae, and tarsi dark brown. Head with small, hemispherically prominent eyes, their diameter equals minimum interocular distance, antennae flat, slender, acutely serrate, apical process of antennomere 3 about half length of its body, further antennomeres similar in shape. Pronotum transverse, with apparent bulge in basal third, almost parallel-sided between bulge and posterior angles, elytra with weak but regular secondary costae and mostly regular, quadrate, small cells.

##### Distribution.

New Guinea, Elelim region.

##### Etymology.

The specific name refers to the type locality.

##### Remark.

*Eniclases versicolor* Kleine, 1926 from an unspecified locality in New Guinea was studied (Holotype, male, ‘Neuguinea, Coll. Kraatz’ deposited in DEIM). *Eniclases versicolor* is similar in general appearance, but differs in large male eyes. The female specimen of *Eniclases versicolor* from the Jayapura district cited by Bocak and Bocakova (1991) might be conspecific with *Eniclases variabilis* or *Eniclases elelimensis* and these species might have allopatric distribution.

#### Eniclases bokondinensis sp. n.

Taxon classificationAnimaliaColeopteraLycidae

http://zoobank.org/C10E3541-F9FB-4C01-8570-9F7568B3BBD7

Figs 17 , 18 , 32

##### Material examined.

Holotype. Female (BM0095), Indonesia, Irian Jaya, Bokondini, 1900 m, Nov.–Dec. 1991 (LMBC). Paratypes. 2 females (BM0092, 94), the same data as the holotype (LMBC).

##### Diagnosis.

*Eniclases bokondinensis* has a characteristic combination of the black pronotum and light yellow elytra with dark colored apex (Figs 17–18). The shape of antennae and the relative size of eyes are similar to those of *Eniclases variabilis* and *Eniclases elelimensis* (Table 1, Figs 32, 38, 39). The co-mimics of *Eniclases bokondinensis* are large-bodied and this species has a larger body than its closest relatives (Fig. 1, Table 1).

##### Description.

Female. Body length 9.2 mm, head, antennae, thorax, and abdomen dark brown to black, elytra pale yellow in humeral half, gradually infuscate to apex (Figs 17–18), trochanters and basal parts of femora brown, rest of legs black. Head with small, hemispherically prominent eyes, their diameter 0.72–0.82 interocular distance, antennae flat, slender, acutely serrate, apical process of antennomere 3 about half length of its body, further antennomeres similar in shape. Pronotum transverse, with weak bulge in basal third, elytra with weak but regular secondary costae and mostly regular, subquadrate cells (Figs 17–18).

##### Distribution.

New Guinea, Bokondini region.

##### Etymology.

The specific name refers to the type locality.

#### Eniclases variabilis sp. n.

Taxon classificationAnimaliaColeopteraLycidae

http://zoobank.org/31A354C9-5025-44C6-878A-5D411699CEB6

Figs 20–29 , 38 , 47

##### Material examined.

Holotype. Male (BM0054), Indonesia, Irian Jaya, Elelim, path to Apalapsili, 600 m, Nov.–Dec. 1991 (LMBC). Paratypes. 1 male, 3 females (BM0048–49, 53, 55), same data as the holotype; 7 males, 8 females (BM0025–32, 34–36, 86, 88, 90–91), Indonesia, Irian Jaya, Bokondini, 1300 m, Nov.–Dec. 1991; 4 males, female (BM0063–67), Indonesia, Irian Jaya, Dombomi, Lower Pass valley, 1200 m; 2 males (BM0008, 12), Indonesia, Irian Jaya, Sentani, Cyclops Mts., 300 m, Nov.–Dec. 1991 (LMBC).

##### Diagnosis.

*Eniclases variabilis* is a widespread, highly polymorphic species and resembles in general appearance several distinct, sympatric mimetic types. The color forms are illustrated in Figs 20–29. Similarly colored individuals of *Eniclases niger* differ in the large eyes (1.17–1.28 times larger than eye distance) and acutely serrate antennae of *Eniclases variabilis* (Figs 38, 40). The similarly colored individuals of *Eniclases elelimensis* can only be identified using the DNA data.

##### Description.

Male. Body length 6.6–8.2 mm. Head, antennae, thorax, and abdomen dark black, elytra orange yellow in humeral third, rest of elytra black, transitional zone between bright and dark part of elytra is narrow (Figs 20–29, 38), trochanters brown, rest of legs black. Head with small, hemispherically prominent eyes, their diameter 0.83–0.95 interocular distance, antennae flat, slender, acutely serrate, apical process of antennomere 3 about half length of its body, further antennomeres similar in shape. Pronotum transverse, with weak bulge in basal third, elytra with weak secondary costae and mostly regular, subquadrate cells (Fig. 20).

##### Distribution.

Central North New Guinea.

##### Etymology.

The specific name refers to exceptional variability in coloration.

##### Remark.

*Eniclases variabilis* can be differently colored in various localities. Generally, the dark colored specimens (Fig. 27) were found in higher elevations in Bokondini (1300 m) and Dombomi (1200 m) and bright colored individuals (Figs 22–23, 26) were collected in lower elevations in Elelim (600 m) and Cyclops Mts. (300 m) (Fig. 3).

#### Eniclases pseudoluteolus sp. n.

Taxon classificationAnimaliaColeopteraLycidae

http://zoobank.org/9E3094C1-2958-4E4B-9D02-C58FCEDC7594

Figs 36 , 48

##### Material examined.

Holotype. Male (BM0084), Indonesia, Irian Jaya, Maibri vill., Arfak Mts., 1600 m, Nov.–Dec. 1991 (LMBC).

##### Diagnosis.

*Eniclases pseudoluteolus* belongs to the group of uniformly yellow species which additionally includes *Eniclases luteolus* Waterhouse, 1878, *Eniclases nigriceps* Bocak & Bocakova, 1991, *Eniclases fuscicornis* Bocak & Bocakova, 1991, and *Eniclases pallidus* Bocak & Bocakova, 1991. Most of them have large eyes (Bocak and Bocakova 1991). Two species, *Eniclases robustus* Bocak & Bocakova, 1991 and *Eniclases proximus* Bocak & Bocakova, 1991 have the similar size of eyes with *Eniclases pseudoluteolus*. These species differ from *Eniclases pseudoluteolus* in the slender antennomere 3 and light colored thorax (antennomere of *Eniclases pseudoluteolus* as in Fig. 36). The similar species occur only in Eastern New Guinea in the vicinity of Wau and Mt. Hagen (Fig. 2).

##### Description.

Male. Body length 9.3 mm, head, apical antennomeres, thorax, and abdomen dark brown to black, pronotum and elytra yellow, trochanters brown, rest of legs black. Head with small, hemispherically prominent eyes, their diameter 0.90 interocular distance, antennae flat, acutely serrate, antennomere 3 triangular, wide, its apical process short (Fig. 36), further antennomeres similar in shape, becoming slenderer to apex of antennae. Pronotum transverse, with weak bulge in basal third, elytra with weak secondary costae and mostly regular, subquadrate cells.

##### Distribution.

New Guinea, Arfak Mts.

##### Etymology.

The specific name refers to the similarity with *Eniclases luteolus*.

#### Eniclases niger sp. n.

Taxon classificationAnimaliaColeopteraLycidae

http://zoobank.org/645B2839-AAD9-4B8A-9741-51C0CB994AEF

Figs 15 , 16 , 40 , 49

##### Material examined.

Holotype. Male (BM0059), Indonesia, Irian Jaya, Elelim, path to Apalapsili, 600 m (LMBC). Paratypes. 3 males (BM0058, 60–61), same data as the holotype; 2 males, 1 female (BM0033, 87, 89), Indonesia, Irian Jaya, Bokondini, 1300 m, Nov.–Dec. 1991 (LMBC).

##### Diagnosis.

*Eniclases niger* is polymorphic in coloration and can be uniformly black or can have the brightly colored pronotum and scutellum (Figs 15–16). The latter specimens resemble some individuals of *Eniclases variabilis* sp. n. when they occur in the same locality. These two species differ in the relative size of eyes (Table 1).

##### Description.

Male. Body length 9.2–11.6 mm (Table 1), uniformly dark colored, only trochanters and bases of femora brown (Fig. 16). Head with large hemispherically prominent eyes, their diameter 1.17–1.28 times distance between eyes. Antennae serrate, with apical process about a third of antennomere stem (Fig. 40). Pronotum transverse, with almost straight lateral margins, lateral bulge inconspicuous, elytra with weak secondary costae and in some parts with irregular cells.

##### Distribution.

New Guinea, Central Mountains.

##### Etymology.

The specific name refers to body coloration.

##### Remark.

*Eniclases niger* has two forms (Figs 15, 16). The specimens from Bokondini have the brightly colored pronotum and scutellum (Fig. 16), the individuals from other localities are uniformly dark colored (Fig. 15).

#### Eniclases similis

Taxon classificationAnimaliaColeopteraLycidae

Bocak & Bocakova, 1991

Figs 13 , 14 , 37 , 50

Eniclases similis Bocak & Bocakova, 1991: 210.

##### Material examined.

10 males, 2 females (BM0003–4, 11, 13–14, 19–24, 37), Indonesia, Irian Jaya, Sentani, Cyclops Mts., 300 m, Nov.–Dec. 1991 (LMBC).

##### Diagnosis.

*Eniclases similis* differs from the similarly colored individuals of *Eniclases divaricatus* in large eyes (Table 1) and from *Eniclases bicolor* in the gradual transition between the dark and bright parts of elytra (Figs 10, 13–14). Despite distant relationships between *Eniclases similis* and *Eniclases bicolor* (Fig. 1) there is no clearly defined morphological character available for their identification.

##### Redescription.

Male. Body length 7.5–9.7 mm, pronotum, humeral part of elytra, and legs yellow to orange, metathorax, abdomen, apical part of elytra, and sometimes antennae dark brown to black (Figs 13–14). Head with moderately large eyes, their diameter 1.17–1.28 times longer than interocular distance, antennae slender, acutely serrate, with apical process of antennomere 3 about third of antennomere stem. Pronotum transverse, with weak lateral bulge in posterior third, elytra with weak, but straight secondary costae and regular, subquadrate cells.

##### Distribution.

New Guinea Highlands.

##### Remark.

*Eniclases similis* has two extreme forms in the extent of the bright part of elytra (Figs 13, 14) and transitional forms between these. The similar color patterns are present in sympatrically occurring species of *Trichalus*. The species is identified as *Eniclases similis* due to the absence of any morphological difference when compared to the holotype of *Eniclases similis*. The type locality of *Eniclases similis* is Karimui in Eastern New Guinea and identity of the species needs further investigation.

## Discussion

The taxonomy of *Eniclases* has been based solely on the morphological species concept, which depends on the presence of identifiable diagnostic traits (Kleine 1926, Bocak and Bocakova 1991). Such species delimitation is difficult as these beetles are morphologically similar and their diagnostic characters are limited to the relative size of eyes, the shape of antennomeres and body coloration (Figs 6–42). Net-winged beetles are unpalatable and the advergence to the similar body shape and coloration has been shown in other metriorrhynchines (Bocak and Yagi 2010). Therefore, we suggest that *Eniclases* are similarly selected to resemble sympatrically occurring net-winged beetles. The high degree of resemblance can be demonstrated by similar color patterns of sympatrically occurring and distantly related *Eniclases similis* and *Eniclases divaricatus* (Figs 1, 6, 13). Already the morphology based revision of *Eniclases* by Bocak and Bocakova (1991) pointed to a high degree of uncertainty in species delimitation and suggested that some species, such as size and color variable *Eniclases luteolus*, probably represent a group of morphologically similar species whose status cannot be resolved morphologically.

Morphological and DNA sequence diversification was investigated in a clade of 14 *Eniclases* net-winged beetles from the western part of New Guinea, delimited as separate species using mtDNA haplotypes, morphological characters and biogeography (DeSalle et al. 2005). We hypothesize that these clusters of individuals represent biological species and are reproductively isolated. Several species are unique in coloration, the shape of antennae, and size of eyes and they were inferred consensually as separate species by all DNA-based analyses: *Eniclases pseudoapertus* (the closest interspecific match at 10.62%), *Eniclases divaricatus* (11.02%), *Eniclases apertus* (6.20%), and *Eniclases pseudoluteolus* (9.26%). The genetic distances between these species and their closest relatives are higher than generally accepted intraspecific genetic variation (Hebert et al. 2003, Meier et al. 2006), the posterior delimitation probabilities for the branches leading to these species were all over 90% except the branch of *Eniclases divaricatus* (69%).

A much higher degree of uncertainty was found in pairs of closely related terminals, which were refused as putative species by various methods (Fig. 1). We can delimit broadly defined species as suggested by the barcoding threshold (Fig. 1) and consider internal clades as forms without formal taxonomic delimitation or we can delimit each clade as a separate species. The first approach makes morphological definitions difficult, as at least in some cases, we would have to merge in a single species some individuals with clearly different morphology. Additionally, there are indications that the narrowly defined species are independently selected for different mimetic patterns in various localities and the color patterns supposedly support reproductive isolation (Bocak and Yagi 2010). Therefore, we prefer to split the closely related clades into separate species when they are supported by clear morphological difference (e.g. size of eyes as *Eniclases similis* and *Eniclases niger* or *Eniclases infuscatus* and *Eniclases bicolor*); they occur allopatrically, belong to different mimicry rings and simultaneously differ in the sequence of *cox1* mtDNA (e.g., *Eniclases bokondinensis* and *Eniclases elelimensis*). In one case, the sister clades are defined by divergent DNA sequence and distribution, but no morphological divergence was identified (*Eniclases tikapurensis* + *Eniclases* sp. A, Fig. 1). Below, we discuss reasons for species delimitation in detail.

The pair of *Eniclases infuscatus* and *Eniclases bicolor* represents sister species which differ in coloration (Figs 10–11) and the relative size of female eyes (Table 1). They were marginally inferred as separate species using the bPTP model (PP 0.47 and 0.52 versus 0.48 for the branch merging these two entities), but clustered together as a putative single species using the distance method (0.09–1.08% distance). Therefore, based on coloration, size of eyes, and genetic difference, we consider these subclades as separate species.

Similarly, *Eniclases bokondinensis* and *Eniclases elelimensis* represent genetically close species with different color patterns (Figs 17–19). The bPTP model merged them in a single putative species and the distance method merged both of them with *Eniclases variabilis*. Both species are known from the Central Mts.; *Eniclases bokondinensis* from the mountain region north of Bokondini (~1900 m a. s. l.) and *Eniclases elelimensis* from Elelim (600 m a. s. l., Fig. 2) about 80 km away. We suppose that in this case the memberships in different mimetic rings play a role in genetic differentiation between these two species (Bocak and Yagi 2010). Species with the *Eniclases elelimensis* color pattern occur in low elevations and have never been collected in high mountains in the Bokondini area. This pattern was recorded as high as 1300 m a. s. l. in, but in a biotope different from mountain forests where *Eniclases bokondinensis* occurs. We suppose that these species occur allopatrically despite the proximity of localities and additionally the colonization of high elevations could support their reproductive isolation (Toussaint et al. 2014).

*Eniclases variabilis* is a sister to the *Eniclases bokondinensis* + *Eniclases elelimensis* clade and was identified as a separate entity using the bPTP model. Other methods merged this species with its sister clade (genetic distance, Fig. 1) or a part of it (morphology, see taxonomy section). The reproductive isolation of *Eniclases variabilis* is supported by the sympatric occurrence of genetically differentiated *Eniclases variabilis* and *Eniclases elelimensis* in Elelim. *Eniclases variabilis* is extremely polymorphic (Figs 20–29) and resembles black colored *Eniclases niger*, brightly colored *Eniclases similis* and *Eniclases elelimensis* types, and one color pattern is unique in *Eniclases* and resembles other net-winged beetles (Figs 20–29). No genetic differentiation has been found in their mtDNA and we consider all color forms as a single species.

The clade of *Eniclases similis* + *Eniclases niger* was merged into a single putative species by all DNA based analyses, but they can be identified by morphology and color patterns. *Eniclases niger* has large eyes (diameter/distance ratio 1.17–1.28) and *Eniclases similis* has smaller eyes (1.02–1.15). We suppose that the daytime or evening, eventually night activity of respective species might be the reason for observed morphological differentiation. Additionally, these species belong to different mimetic complexes. *Eniclases niger* is black colored (Fig. 15) or has pronotum bright and elytra completely black (Fig. 16) and *Eniclases similis* is brightly colored. The bright patterns are similar to those of *Eniclases divaricatus* and *Eniclases bicolor* (Figs 6, 10, 13–14) and were recorded also in several *Trichalus* spp. in the respective localities (unpublished data). These sister-species, *Eniclases similis* and *Eniclases niger*, have not been collected sympatrically. *Eniclases niger* occurs in lower mountain localities in the Central Mts. and *Eniclases similis* in low elevations of the Cyclops Mts.

The clade of *Eniclases tikapurensis* and *Eniclases* sp. A split in two subclades, which do not differ in morphology, but they are genetically distant. The levels of DNA distances between *Eniclases tikapurensis* and *Eniclases* sp. A do not agree with the geographical distance of respective localities, when higher differentiation was found between populations from Bokondini and Tikapura (13 km apart) than between localities lying at the rim of the Baliem valley (Yiwika and Tikapura, 35 km apart). The observed genetic differentiation surpasses some cases when separate morphologically divergent sister species are delimited. Due to limited information we postpone formal description of the putative species from Bokondini.

*Eniclases* are variable in coloration (Figs 6–29), but the differences in coloration regardless of their depth do not necessarily mean that they can be used for delimitation of reproductively isolated lineages. We found intraspecific color variability in three species: *Eniclases similis*, *Eniclases niger*, and *Eniclases variabilis* (Figs 13–14, 15–16, 20–29). We ascribe the polymorphism to the advergence to the most common models of other net-winged beetles as described by Bocak and Yagi (2010). Conversely, the color patterns can be used in several species for their delimitation, e.g. in *Eniclases bicolor* and very characteristic *Eniclases bokondinensis*. Molecular data or further morphological characters are generally needed to investigate color polymorphism and to support species limits. Uncorrelated morphological and genetic diversity has been reported in lyponiine lycids (Li et al. 2015) and other beetle families (e.g. Goldberg et al. 2012) and points to the necessary evaluation of all evidence when species are delimited (Jorger and Schrodl 2013). The *cox1* mtDNA fragment alone cannot provide sufficient information on the diversification process, but even these limited data suggest that closely related lineages can develop distant mimetic patterns and start further morphological differentiation, for example in the relative size of eyes. The dated phylogeny suggests that all closely related sister species differentiated in the last million years (Fig. 2).

## Supplementary Material

XML Treatment for Eniclases

XML Treatment for Eniclases pseudoapertus

XML Treatment for Eniclases divaricatus

XML Treatment for Eniclases infuscatus

XML Treatment for Eniclases bicolor

XML Treatment for Eniclases tikapurensis

XML Treatment for Eniclases brancuccii

XML Treatment for Eniclases elelimensis

XML Treatment for Eniclases bokondinensis

XML Treatment for Eniclases variabilis

XML Treatment for Eniclases pseudoluteolus

XML Treatment for Eniclases niger

XML Treatment for Eniclases similis
